# The Multifaceted Roles of Autophagy in Infectious, Obstructive, and Malignant Airway Diseases

**DOI:** 10.3390/biomedicines10081944

**Published:** 2022-08-11

**Authors:** Marianna Carinci, Laura Palumbo, Giulia Pellielo, Esther Densu Agyapong, Giampaolo Morciano, Simone Patergnani, Carlotta Giorgi, Paolo Pinton, Alessandro Rimessi

**Affiliations:** 1 Laboratory for Technologies of Advanced Therapies, Section of Experimental Medicine, Department of Medical Sciences, University of Ferrara, 44121 Ferrara, Italy; 2 Center of Research for Innovative Therapies in Cystic Fibrosis, University of Ferrara, Via Fossato di Mortara, 70, 44121 Ferrara, Italy

**Keywords:** lung disease, lung infectious, autophagy, xenophagy SARS-CoV-2, Respiratory Syncytial Virus, *Mycobacterium tuberculosis*, Cystic Fibrosis, COPD, Malignant Mesothelioma

## Abstract

Autophagy is a highly conserved dynamic process by which cells deliver their contents to lysosomes for degradation, thus ensuring cell homeostasis. In response to environmental stress, the induction of autophagy is crucial for cell survival. The dysregulation of this degradative process has been implicated in a wide range of pathologies, including lung diseases, representing a relevant potential target with significant clinical outcomes. During lung disease progression and infections, autophagy may exert both protective and harmful effects on cells. In this review, we will explore the implications of autophagy and its selective forms in several lung infections, such as SARS-CoV-2, Respiratory Syncytial Virus (RSV) and *Mycobacterium tuberculosis* (Mtb) infections, and different lung diseases such as Cystic Fibrosis (CF), Chronic Obstructive Pulmonary Disease (COPD), and Malignant Mesothelioma (MM).

## 1. Introduction

### 1.1. Autophagy Overview

Autophagy refers to a non-selective degradation process that supports the elimination of damaged organelles and structures to maintain cellular homeostasis. Furthermore, autophagy represents a fundamental system that provides nutrients (amino acids, lipids, and other precursors) for survival during starvation [1]. Based on the different mechanisms used to deliver cargo to the lysosome, autophagy may be classified as macroautophagy, microautophagy, and Chaperone-Mediated Autophagy (CMA). Macroautophagy (hereafter referred to as autophagy) is a cellular process by which cytoplasmic materials are trapped in double membranes, from where they are delivered to lysosomes for degradation [2]; microautophagy is a mechanism where the invagination of the lysosome directly engulfs cytoplasmic contents to degrade them [3]; CMA is a process by which molecular chaperones bind protein targets and bring them to lysosomes for degradation [4].

#### 1.1.1. Macroautophagy

**Figure 1:**
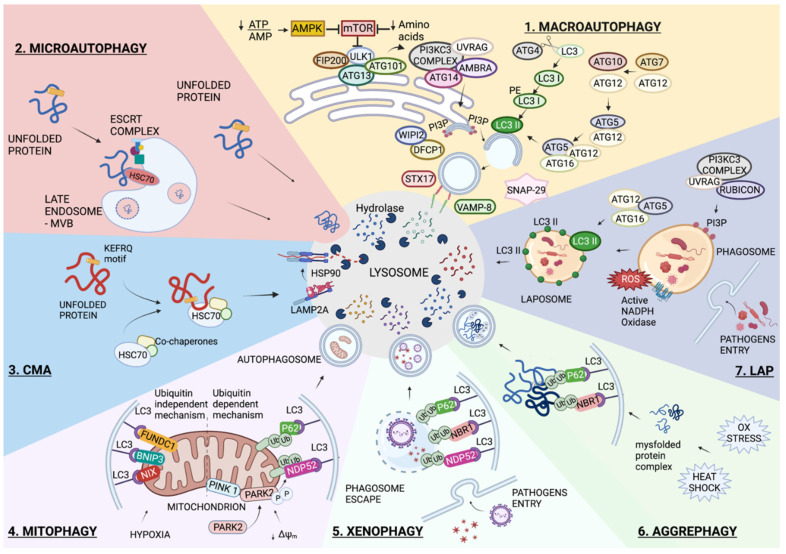
Autophagy overview. Schematic representation of the main autophagic pathways involved in lung infections and diseases. All these degradative pathways have the lysosomal degradation in common. 1. Macroautophagy is the non-selective degradation of intracellular content. The process starts with AMPK activation and/or MTOR inactivation. The recruitment of several complexes composed of ATG proteins orchestrates the formation of a double membrane vesicle called an autophagosome, and the enwrapped content is degraded following its fusion with a lysosome. 2. Microautophagy occurs through the direct invagination of the lysosomal membrane into the lumen. In endosomal microautophagy, the invagination of a late endosome membrane is mediated by the ESCRT complex, and a KFERQ-like motif on the substrate is recognized by HSC70. 3. Similar to endosomal microautophagy, Chaperone-Mediated Autophagy (CMA) requires KFERQ-like motifs on substrates that are recognized by HSC70; however, CMA also requires the LAMP2A lysosomal protein receptor to translocate the unfolded substrate into the lysosome lumen. The selective forms of autophagy require the formation of the autophagosome and differ according to the specificity of the substrate. 4. Mitophagy is the selective degradation of mitochondria, which can occur through the ubiquitin-dependent or ubiquitin-independent pathways. 5. Xenophagy is the selective removal of pathogens. 6. Aggrephagy refers to the degradation of cytoplasmic protein aggregates. 7. A non-canonical form of autophagy is represented by the LC3-Associated Phagophore, LAP. Pathogens are enclosed in a single-membrane vesicle, and ROS generated by NADPH oxidase (NOX) promotes the recruitment of LC3 to the phagosome. The formed LAPosome fuses with the lysosome for content degradation. More details are reported in the main text. Created with BioRender.com.

The autophagy pathway is mainly characterized by four stages: initiation, elongation, maturation, and fusion/degradation. The mechanistic target of rapamycin (mTOR), which is preeminently responsible for autophagy induction, plays a crucial role in sensing nutrient availability. More specifically, amino acid deficiency is one of the main cellular stresses that inhibits mTOR. Under nutrient-rich conditions, the mTORC1 complex inactivates Unc-51-Like Kinase 1 (ULK1) through the phosphorylation of its Ser757, leaving the autophagy at low levels. On the contrary, when there is a lack of amino acids, the inactivated mTORC1 dissociates from ULK1, resulting in ULK1 activation, which phosphorylates the proteins involved in the autophagosome formation [5]. Autophagy activation is also induced by glucose starvation, which is identified by the reduction of the ATP:AMP ratio. The 5′ AMP-Activated Protein Kinase (AMPK) acts as a sensor that inhibits mTORC1, mediating the phosphorylation of the Raptor subunit or directly phosphorylating ULK1, Vacuolar Protein Sorting 34 (VPS34), and Beclin 1 (BECN1) [6]. Following these cellular stress, Autophagic-Related Genes (ATGs) are recruited to a specific site called the phagophore or the isolation membrane, which is best characterized by its localization in the Endoplasmic Reticulum (ER) [7]. The first actors involved in autophagy initiation are the ULK1 complex, composed of ULK1, ATG13, RIB-inducible coiled-coil protein 1 (FIP200), and ATG101, and the only known transmembrane protein ATG9; the latter is required to deliver membranes to the pre-autophagosome structures and autophagosomes. During the initiation step, ULK1 kinase activates the Phosphatidylinositol 3-Kinase Catalytic Subunit Type 3 (PI3KC3) complex, which consists of class III PI3K, VPS34 [8], BECN1 [9], and the general vesicular transport factor (p115) [10]. This complex can be linked either to ATG14 [11] and the Activating Molecule in BECN1-Regulated Autophagy protein 1 (AMBRA1) [12] or to the UV radiation resistance-associated gene protein (UVRAG) [9], which successively generates Phosphatidylinositol 3-Phosphate (PI3P). Then, the WD Repeat Domain, Phosphoinositide Interacting 2 (WIPI2), and Zinc Finger FYVE-Type Containing 1 (DFCP1) are recruited by PI3P to form specific ER domains called “omegasomes” (for their omega, Ω, shape) [13]. The elongation of the autophagosome membrane requires two ubiquitin-like conjugation systems: the conjugation of ATG12-ATG5 and ATG8 proteins (Microtubule-Associated Protein 1A/B Light-Chain 3-LC3s and Gamma-Aminobutyric Acid Receptor-Associated Protein-GABARAPs) conjugated to Phosphatidylethanolamine (PE) [14]. ATG7 activates ATG12 and transfers it to ATG10, which mediates the conjugation to ATG5. At the same time, ATG4 proteases cleave the precursor of LC3 to produce cytoplasmic LC3-I, which is then coupled with PE by interacting with ATG7, ATG3, and ATG12-ATG5, respectively. This lipidation converts the soluble LC3 into the membrane-bound form, thus promoting autophagosome formation [15]. The final step of autophagy is the fusion of autophagosomes with lysosomes, which needs soluble N-ethylmaleimide Sensitive Fusion Protein (NSF) Attachment Receptor (SNARE) proteins. The main autophagosomal soluble SNARE protein is Syntaxin 17 (STX17), which forms a complex together with Synaptosome Associated Protein 29 (SNAP-29) and the lysosomal SNARE Vesicle Associated Membrane Protein 8 (VAMP-8), allowing for the fusion of autophagosomes with lysosomes [16] (Figure 1).

#### 1.1.2. Microautophagy

Microautophagy is the process by which cytoplasmic cargos are directly engulfed through lysosome membrane invagination without the formation of autophagosomes. A particular form of microautophagy, called endosomal microautophagy, has also been reported. This process occurs during MVB formation and requires the Endosomal Sorting Complexes Required for Transport (ESCRTs) [17]. The ubiquitinated proteins located on endosomal membranes are linked to the ESCRT-0 complex, which then assembles the ESCRT-I and -II complexes to make ESCRT-III. The ESCRT-III complex endorses the invagination, constriction, and abscission of membranes. Protein cargo selection is mediated by the chaperone Heat Shock Cognate 71 kDa Protein (HSC70) [18]. In mammals, rapamycin has been reported to activate microautophagy, suggesting that mTORC1 controls ESCRT-0-mediated microautophagy [19] (Figure 1).

#### 1.1.3. Chaperone-Mediated Autophagy (CMA)

Compared with macroautophagy and microautophagy, CMA is a unique pathway in which proteins translocate one by one into the lysosomal lumen [20]. More specifically, CMA begins with the binding of HSC70 [21] to a pentapeptide motif, KFERQ-like, on the substrate protein, thus making the HSC70/substrate protein complex [22]. Subsequently, this complex is delivered to the surface of lysosomes, where it interacts with the cytosolic tail of the Lysosome-Associated Membrane Protein type 2A (LAMP2A) [23]. In particular, the substrate protein needs to be unfolded by HSC70 and its co-chaperones in order to translocate into the lysosomes. This process not only requires a luminal form of HSC70 (lys-HSC70) but also of HSP90, which participates in the transition of the LAMP2A monomer to the multimer stage. The LAMP2A multimer promotes the substrate translocation into the lysosomal lumen, where it is completely degraded by hydrolytic enzymes [24] (Figure 1).

#### 1.1.4. Selective Autophagy

In addition to non-selective autophagy, autophagosomes, under certain conditions, selectively target cytosolic materials. This type of autophagy, termed selective autophagic response, is classified according to the cargo to be degraded: mitochondria (mitophagy), pathogens (xenophagy), protein aggregates (aggrephagy), cilia (ciliophagy), lipid droplets (lipophagy), ER (ER-phagy) [25], etc.

Selective autophagic responses are divided into the ubiquitin-dependent pathway—achieved by Sequestome-Like Receptors (SLRs) such as Sequestome (p62/SQSTM1), Neighbor of BRCA1 Gene 1 Protein (NBR1), optineurin (OPTN), Calcium Binding And Coiled-Coil Domain 2 (NDP52), and Tax1 Binding Protein 1 (TAX1BP1), and the ubiquitin-independent pathway—mediated by receptors that include Bcl-2 Interacting Protein 3 (BNIP3), Bcl-2 Interacting Protein 3 Like (BNIP3L/NIX), and FUN14 Domain Containing 1 (FUNDC1), which act as bridges between the substrate and ATG8, located on autophagosomes [26].

##### Mitophagy

Despite the mitochondria-derived vesicles being degraded by lysosomes, several studies highlight the importance of mitophagic mediators that interact with LC3 [27]. In fact, the targeted mitochondria are recognized by autophagosomes through LC3 adapters, mainly in (i) a ubiquitin-dependent pathway and (ii) a ubiquitin-independent pathway (Figure 1).

(i). Mitochondrial depolarization causes the accumulation of mitochondrial PTEN Induced Kinase 1 (PINK1) on the Outer Mitochondrial Membrane (OMM) [28,29]. The activation of PINK1 through its auto-phosphorylation starts mitophagy in two parallel processes: (a) the phosphorylation of ubiquitin at serine 65 and (b) the phosphorylation of Parkin RBR E3 Ubiquitin Protein Ligase (PARK2) [30]. The interaction of phosphorylated PARK2 with phospho-ubiquitin on mitochondria results in PARK2 activation, which is responsible for the ubiquitination of OMM proteins [31,32]. This process permits the recruitment of autophagic receptors to the OMM, which links ubiquitylated proteins with autophagosomes via their ubiquitin-binding domains and LC3-Interacting Region (LIR) motifs, respectively [33].
(ii). The LC3 receptors on the mitochondrial membrane directly bind to LC3 and play an important role in the recruitment of damaged mitochondria to the autophagosomes. These receptors, including NIX, BNIP3, and FUNDC1, induce mitophagy under hypoxia conditions [34,35,36]. Bcl-2 like 13 (Bcl-2L13) and FKBP Prolyl Isomerase 8 (FKBP8) are located on the OMM and mediate mitophagy by interacting with LC3, via the LIR motif [37] and via LC3A [38], respectively.

##### Xenophagy

Xenophagy is a specialized autophagic response that plays a fundamental role in the clearance of invading pathogens [39]. After the infection of mammalian cells, pathogens are restricted to vacuoles; however, their growth may disrupt the vacuoles, leading to an invasion of the host cytoplasm [40]. The ubiquitin (Ub)-chains act as a degradation target of pathogens and are subsequently recognized by different autophagic receptors, including p62, NBR1, and NDP52. These receptors then link the Ub-decorated membrane to LC3 to entrap the pathogens in autophagosomes for degradation [41] (Figure 1).

##### Aggrephagy

The production of misfolded proteins generally occurs during protein synthesis due to cellular stresses such as oxidative stress and heat shock. Misfolded proteins, which are not eliminated, can aggregate and consequently compromise cellular homeostasis. A selective type of autophagy, called aggrephagy, acts as a quality control mechanism that removes aggregated proteins [42]. In particular, the first step is the ubiquitination of protein aggregates, which are then identified by several receptors, including p62 and NBR1 [43]. These receptors link the ubiquitinated protein aggregates with autophagosomes by simultaneously binding to ubiquitin chains and the LC3-family of proteins [44] (Figure 1).

#### 1.1.5. LC3-Associated Phagocytosis (LAP)

LC3-associated phagocytosis (LAP) is a novel, non-canonical autophagic response of phagocyte cells that emerges to control infection and the immune response; it is involved in the clearance of apoptotic cells, in a process called efferocytosis [45]. Compared to canonical autophagy, the formation of LAPosome, contrary to classical autophagosome, is formed by a single lipid layer and does not require the ULK1 complex nor mTOR inhibition. In addition, LAP is independent of ATG14, and it requires the PI3KC3 complex, UVRAG, and RUN And Cysteine Rich Domain Containing Beclin 1 Interacting Protein (Rubicon) [46,47]. ATG5-ATG12-ATG16 is important for the conjugation of LC3 on the phagosomal membrane [48,49]. Furthermore, the unique property of LAP is the requirement of Reactive Species of Oxygen (ROS) production by the NADPH oxidase-2 (NOX2) complex. This complex is expressed in phagocytes [50] and plays an important role in stabilizing Rubicon, which increases the superoxide anion production [51] (Figure 1). Despite LAP being considered a non-canonical autophagy, more investigations are needed to define the molecular mechanism underlying its function.

## 2. Autophagy in Lung Infections

Autophagy and its selective form, xenophagy, play an important role in the response to pathogen infections by sequestering invading pathogens for lysosomal degradation. To date, host-directed therapies targeting autophagy in order to control pathogen infection are increasingly attracting interest. In this section, we will analyze the role of autophagy in different pathogen infections by emphasizing the targeting of autophagy in the resolution of infection.

### 2.1. Autophagy in SARS-CoV-2 Infection

Severe Acute Respiratory Syndrome Coronavirus 2 (SARS-CoV-2) is a disease associated with a new viral strain that appeared in 2019; it belongs to the Orthocoronavirinae subfamily and has the ability to infect humans. Being an RNA-based virus, it is prone to easily change over time by introducing mutations, which can modify its infectious capacity, virulence, and downstream molecular pathways once the virus enters the cells.

#### 2.1.1. Exploiting Autophagy by SARS-CoV-2

All publications available from 2020 agree with the fact that SARS-CoV-2 modifies the autophagic pathway of infected cells, which then creates an escape mechanism against humans’ defenses [52]. However, how SARS-CoV-2 interferes with autophagy is apparently controversial. Most research agrees with the block of autophagic flux operated by SARS-CoV-2, which has been observed in host epithelial, immune, and respiratory cells [53,54], rather than the stimulation of autophagy or mitophagy to sustain high levels of replication, as reported by a limited number of studies [55,56].

**Figure 2:**
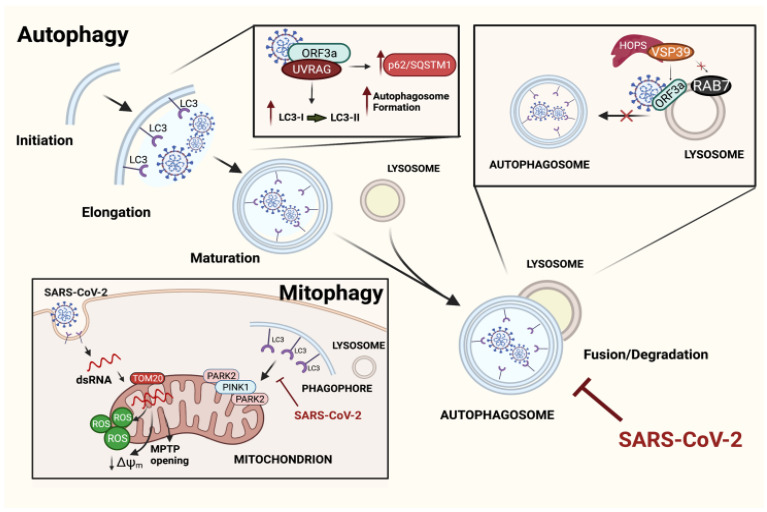
Mechanisms of the SARS-CoV-2 evasion of autophagic pathways. Illustration depicting principal mechanisms developed by SARS-CoV-2 in order to use autophagy pathways for its own benefits. Diagonally, the main steps of the autophagic process are represented. The viral ORF3a interacts with UVRAG at high affinity, undermining the link UVRAG-BECN1 and promoting an accumulation of autophagosomes, which never fuse with lysosomes. Another mechanism through which ORF3a inhibits the autophagosome–lysosome fusion is by binding VPS39 of the HOPS complex, thus preventing the RAB7–VSP39 interaction. Even mitophagy has been reported to be affected by SARS-CoV-2. Indeed, once inside the cell, the viral dsRNA enters the mitochondria, thus leading to mitochondrial dysfunctions that promote PINK1/PARK2-dependent mitophagy. However, the process never ends as the virus blocks the incorporation of the mitochondria into autophagosomes, improving viral replication. Created with BioRender.com.

Based on the earlier literature, SARS-CoV-2 takes advantage of several proteins to manipulate autophagy by inducing a block in its final stages. The Open Reading Frame (ORF)3a and ORF7a are two of the most studied proteins of the viral genome, and their study has led to similar results by independent groups. Among all the 28 viral proteins, their expression induced the significant conversion of LC3-I into LC3-II and the greater stability of the p62 protein, suggesting that the fusion step leading to autophagosome maturation had been blocked by SARS-CoV-2 infection [53]. Indeed, the infection should block the autophagosome–lysosome fusion and consequently degenerate into an incomplete and inefficient autophagic response. The host target of ORF3a was found in UV Radiation Resistance-Associated Gene (UVRAG), with a strong binding affinity; the occurrence of the ORF3a–UVRAG interaction reshaped other essential physiological protein–protein interactions in infected cells, which presented defects in the UVRAG-Beclin1 bindings, thus promoting the associated regulation of the autophagosome formation [53] (Figure 2). This feature is one of the peculiarities discovered to be in charge of this new virus; it has been acquired as a gain of function and is not present in CoV infections from the early 2000s.

Similar results were obtained by Zhang Y and co-workers [57], who identified additional motifs in the structure of the virus for interactions with new host targets. Indeed, more importance was ascribed to the transmembrane domain and the C-terminus (instead of N-terminus) of SARS-CoV-2 in blocking autophagy by binding the Vacuolar Protein Sorting (VPS) 39, which is part of the HOPS molecular complex that is indispensable for the tethering of both autophagosomes and lysosomes. Even in this case, the formation of the ORF3a–VPS39 complex blocks autophagy and affects the Ras-Associated Protein 7 (RAB7)–VSP39 physiological binding, which is significantly reduced (Figure 2).

Other proteins, such as ORF7a, Nsp15, E, and M, modulate autophagy, although to a minor extent. Whether ORF7a and E act in a similar way to ORF3a needs to be validated. The M and Nsp15 proteins share different mechanisms that appear to interfere with the de novo activation of autophagy [54].

One of the last studies in the field reported the localization of the dsRNA of the virus in the mitochondria of infected cells a few hours after infection; the OMM protein TOM2O was suggested to be responsible for transporting the viral dsRNA into the organelle. Once in the mitochondria, SARS-CoV-2 induces several mitochondrial dysfunctions, including the alteration of membrane potential, morphology, and mitochondrial permeability transition [58,59]; all mitochondrial events are compatible with cell death in chronic conditions [60]. Furthermore, the activation of PARK2 and PINK1-dependent mitophagy has been documented, and it is triggered by hosting cells as a mechanism of quality control in order to counteract the accumulation of damaged mitochondria; however, the virus is capable of blocking the incorporation of mitochondria into autophagosomes. This process has been demonstrated to improve the replication rate of the virus [60] (Figure 2).

A similar study identified all these impaired processes (autophagy and mitophagy) as the cause of inflammasome activation and the development of a cytokine storm that induced pyroptosis and affected multiple organs, primarily the lung [61].

On the contrary, and as mentioned above, some studies reported the activation of autophagy and mitophagy by the coronavirus as mechanisms to be exploited for its replication. In one of them, the protein ORF10 was able to induce mitophagy by binding the mitochondrial receptor BNIP3L/NIX. A mitophagy burst induced the degradation of the Mitochondrial Antiviral-Signaling Protein (MAVS), a protein that is involved in immune defense and promotes the downregulation of Interferon (IFN)-I signaling [55]. On the other hand, Shang C. et al. proposed a double mechanism for the control of autophagy modulation by SARS-CoV-2: the induction of the first stages of autophagy in different animal models with the inhibition of the Akt–mTOR axis and the overexpression of the ULK-1 and VPS34-BECN1 interaction, but with concomitant blocks of the autophagosome–lysosome complex [56].

#### 2.1.2. Targeting Autophagy in SARS-CoV-2 Infection

Since most of the literature agrees that SARS-CoV-2 escapes from autophagy clearance, the improvement of the autophagic flux in the host organism may determine a strategy to counteract the infection from the virus. Several drugs have been proposed to fight SARS-CoV-2, but most of them have already been abandoned due to controversial results, a low degree of safety, and documented effects on autophagy, but not on the autophagic flux [62].

In our opinion, the most interesting drugs to be considered in the fight against coronavirus infection, and for which there are more advanced studies, are some antidepressants. First, their consumption is well-known to modulate the autophagic flux [63,64], and second, there are many retrospective studies available for analysis, in which patients who are affected by SARS-CoV-2 simultaneously consume antidepressants. Very recently, a meta-analysis found that patients affected by SARS-CoV-2 and were under treatment with antidepressants had a reduced risk of intubation and death [65]. Just to name a few of these drugs, several trials have been registered and are currently carrying out analyses of the effects of Fluvoxamine, Fluoxetine, and Donepezil. Moreover, besides inducing autophagy, they are also agonists of the sigma-1 receptors [66], a molecular target involved in ER stress and cytokine-mediated inflammation.

Ivermectin and Niclosamide [67] are two additional drugs recently proposed to fight SARS-CoV-2. They induce autophagic flux [68] by targeting the negative regulator of BECN1, the S-Phase Kinase Associated Protein 2 (SKP2), enhancing LC3 lipidation and thereby reducing SARS-CoV-2 replication by approximately 43% [67]. Among other autophagy enhancers, it has recently been proposed that INF-alpha 2b triggers the accumulation of autolysosomes in cells, which is induced by the modulation of BECN1 and LC3-II expression [69]. Lopinavir and Ruxolitinib are able to induce autophagosome formation, therefore downregulating the mTORC1 pathway, while the Tocilizumab antibody has been identified to revert the autophagic degradation [70] and limit the action of IL-6, thus mitigating the inflammation.

Of course, these are not yet specific treatments against SARS-CoV-2; these drugs have several other roles (although beneficial), such as the modulation of the Bcl-2 family of proteins in developing cell death, the downregulation of inflammation in response to the cytokine storm, and more importantly, they are still under investigation in clinical trials.

### 2.2. Autophagy in Respiratory Syncytial Virus (RSV) Infection

Respiratory Syncytial Virus (RSV) is a non-segmented negative-sense single-stranded enveloped RNA virus belonging to the Orthopneumovirus genus [71]. It may be classified into two groups that present distinct surface antigens: RSV-A and RSV-B. It is considered as one of the most important respiratory pathogens causing relevant clinical manifestations in both the lower and upper respiratory tracts of infants and children [72]. The entry mechanism of RSV on target cells depends on several RSV proteins and includes different cellular types [73]. Once the epithelial cells of the upper respiratory tract are infected, RSV infection may spread to the lower respiratory tract where it could induce pulmonary inflammation, neutrophil infiltration, mucus plugging, and consequently, airway obstruction [74]. In the past years, several attempts have been made to develop possible pharmacologic therapies; however, to date, no effective and specific treatment for curing RSV is available. A better knowledge of the molecular mechanism involved in the viral infection and the host–immune response would be useful for the identification of possible drug targets in order to develop specific therapies.

#### 2.2.1. Autophagy Activation in Intracellular Elimination of RSV

Upper airway epithelial cells have been reported to be the primary site of RSV infection [74]. RSV infection of the host leads to the activation of the immune system through its recognition of viral Pathogen-Associated Molecular Patterns (PAMPs). As a result, a cascade of proinflammatory cytokines and antiviral interferons is stimulated [75]. Dendritic cells (DCs) play an important role in antigen recognition, processing, and presentation. Once activated, DCs migrate to lung-draining lymph nodes (LDLNs), promoting T cell activation via costimulatory marker presentation, proinflammatory cytokine release, and antigen presentation. Interestingly, autophagy has been shown to be involved in the regulation of the maturation and differentiation mechanisms of DCs. Indeed, several studies have suggested that RSV infection leads to an autophagy-mediated antiviral adaptive immune response in infected DCs. This occurs through the increased MHC II as well as CD80 and CD86 expression, which is required for antigen presentation and the activation of T cells [76]. Lukacs’s group published comprehensive works in which the role of RSV in the induction of autophagy and the consequent activation of immune response was shown in DCs obtained from infected mice [77,78]. Clinical manifestations of primary RSV infection involved neutrophil infiltration and excessive mucus production, resulting in airway obstruction [79,80]. Moreover, experimental evidence has showed that IL-17a regulates neutrophil infiltration, mucus hypersecretion, and the suppression of CD8 T cell responses in RSV infection [81,82]. Interestingly, the role of autophagy in the blocking of pathology progression through the impairment of IL-17a production has been demonstrated in an autophagy deficiency mouse model [83]. Additionally, macrophages play a fundamental role in the immune response following the entry of a pathogen. Among the different cytokines involved in immune response, IFN-β is one of the major players driving virus clearance. It has been reported that macrophages showed an increased IFN-β-induced antiviral response following RSV infection in order to block viral replication through autophagy-triggered TGF- β and SMAD-2/3 signaling [84].

#### 2.2.2. Exploitation of Autophagy by RSV

Autophagy is an antiviral immune mechanism for maintaining cellular homeostasis and is utilized by infected cells to incorporate and degrade viral components inside autophagolysosomes. However, RSV can revert the physiological autophagy mechanism for its own survival.

Recently, the important role of autophagy in the RSV viral proliferation has been demonstrated in both in vivo and in vitro models [85]. Coherently, a recent study confirmed this correlation, showing that the inhibition of mTORC1 by rapamycin in RSV-infected A549 cells induced the increase of RSV viral proteins and the production of infectious progeny virus [86]. RSV induced the AMPK-mTOR pathway and ROS-dependent autophagy in both HEp-2 and A549 cells for its own replication. Accordingly, the knockdown of key molecules in the autophagic machinery suppressed RSV replication [85]. An alternative way to induce autophagy by RSV infection is linked to NS1, a structural viral protein involved in the evasion mechanism of immunological surveillance in autophagy and viral survival [87,88]. Indeed, it has been suggested that RSV, through NS1, induced autophagy through the mTOR-S6KP70 pathway in order to prevent both the activation of apoptotic mechanism and the release of pro-inflammatory cytokines, thus promoting viral survival [89]. More importantly, another RSV structural protein, NS2, has recently been demonstrated to stabilize Beclin1, preventing its proteasomal degradation and thus inducing autophagy upon RSV infection [90]. Therefore, autophagy may be considered as an escape mechanism used by RSV to improve its proliferation and survival. The involvement of autophagosomes in viral replication was also demonstrated in RSV-infected bronchial cells, where the treatment with nanosized particles prior to infection with RSV increased the efficiency of viral replication [91]. The mechanism through which RSV exploits autophagy could reside in the blockage of autophagosome degradation. According to a recent work, the fusion of autophagosomes and lysosomes does not take place. Das and co-workers assessed the role of IL-22 in reverting the blockade of the autophagic mechanism induced by RSV in both human airways and alveolar epithelial cells [92]. Therefore, according to the author’s view, the infection of lung epithelial cells by RSV initially triggers the formation of the autophagosome but subsequently blocks its fusion with the lysosome, thus preventing the formation of autophagolysosomes, which have been proven to be harmful to viral survival. However, it has not yet been specifically demonstrated how RSV is able to block the autophagic flux. This hypothesis would appear to be in contrast to the work of Li and colleagues [85], in which the activation of autophagic flux was found in infected HEp-2 cell lines. The use of two different cell lines, such as HEp-2 and A549, as an infection model could justify this apparent discrepancy. In addition, Li et al. observed that RSV induced mitophagy, which allowed for a lower release of cytochrome c in the cytosol and the consequent reduction of apoptosis following viral infection. However, further analyses involving the use of specific markers that can selectively confirm this hypothesis would be required.

RSV re-infections are common, as demonstrated by the low number of RSV-specific memory T cells in RSV patients of all ages, which is probably due to a deficit in the memory response induced by RSV [93,94,95]. Since mTOR is involved in the differentiation of CD8 T cells from effectors into memory cells [96,97,98], RSV acts on mTOR in order to escape from this host protective immune response mechanism. Indeed, it has been reported that rapamycin increased the frequency of RSV-specific CD8 T cells, therefore confirming that RSV-induced mTOR phosphorylation is essential for an escape mechanism in the RSV-specific memory differentiation of CD8 T cells [98].

Thus, as demonstrated by the studies reported so far, autophagy plays a changing role according to the target cells. Indeed, at the epithelial level, RSV may induce a blockage of the final phase of the autophagic process, which would create a comfortable environment for viral replication. At the same time, RSV activates mitophagy, which leads to a decrease in the death of infected cells and the consequent worsening of the disease. In parallel, autophagy in immune system cells, such as DCs and macrophages, plays an opposite role, enhancing the response of the immune system in its defense against the entry of pathogens.

#### 2.2.3. Targeting Host Autophagy to Counteract RSV Infection

Given that RSV is one of the most important and prevalent causes of hospital admission and death from acute lower respiratory tract infection in children [99], the development of an appropriate therapy for the treatment or prevention of RSV infection appears to be necessary. In the current state, there are only two RSV antiviral drugs approved by the FDA: Palivizumab and Ribavirin. However, due to the side effects, high cost, and mild efficacy, these two monoclonal antibodies are mainly used as prophylaxis [100,101,102]. Certainly, viral entry and replication are the most promising targets in effective therapies. Starting from these assumptions and given the importance of autophagy in the regulation of viral proliferation and lung pathology progression, the possible use of specific drug-regulating autophagy may be considered as a novel therapeutic approach that would contrast RSV progression, therefore reducing the gravity of its pathological features. Since autophagy plays a different role depending on the RSV-infected target cells, in the same way, several drugs should have different effects on target cells to contrast viral progression.

It has been shown that the administration of chloroquine, a typical inhibitor of autophagy, in a mouse model led to the inhibition of human Respiratory Syncytial Virus A (long strain) replication [103], which makes chloroquine a potential drug for the therapeutic treatment of RSV infection. RSV infection is involved in the subsequent development of clinical manifestations such as asthma [104]. It has been observed that Qingfei (QF), a traditional Chinese medicine used to treat asthmatic patients, is involved in the inhibition of RSV replication, in the reduction of airway hyperresponsiveness, and in mucus hypersecretion in RSV-infected asthmatic mice [105]. Interestingly, a more recent study demonstrated that QF can prevent the asthma exacerbation by inhibiting autophagy via the mTOR signaling pathway in both in vivo and in vitro RSV-infected asthmatic models [106]. Wuhu decoction has recently been reported to significantly reduce lung lesions, chronic airway inflammation, and airway remodeling in an RSV-induced asthmatic mice model through the upregulation of autophagy via AMPK/ULK1 signaling [107].

The root of *Scutellaria baicalensis*, widely used as an antiviral Chinese herbal medicine, contains several bioactive compounds such as Wogonin and Baicalin, which are associated with antiviral action, the reduction of inflammatory cell infiltration, and lung injury in several RSV-infected models [108,109]. More importantly, the molecular mechanisms of all these compounds involve autophagy [110]. It is therefore possible to hypothesize that the antiviral action of these molecules may depend on the regulation of the autophagic mechanism.

Resveratrol, a natural compound used in the treatment of cancer for its ability to induce autophagy [111,112], has also been associated with the improvement of airway inflammation and hyperresponsiveness symptoms in both an RSV- infected mice model and RSV-infected epithelial cells [109,113,114,115].

Although it has been a long time since the virus first appeared, no effective therapeutic remedy has been found so far. Over the years, progresses have been made in trying to understand the mechanism used by RSV to infect and proliferate within the host organism. Autophagy plays important roles in RSV infection. Indeed, its involvement in viral proliferation in infected epithelial cells has been demonstrated, as well as its role in blocking the progression of the disease by enhancing the activation of the immune system cells. These findings suggest that autophagy could be an effective therapeutic target against RSV infections.

### 2.3. Autophagy in Mycobacterium Tuberculosis (Mtb) Infection

Tuberculosis (TB) is a potentially serious and contagious infection. The causative agent of tuberculosis is the intracellular pathogen Mtb, also known as Koch’s bacillus [116]. The infection by Mtb of extra pulmonary sites has been reported [117]; however, the organ mainly affected is the lung, making pulmonary disease the most common clinical presentation. Living Mtb is spread from one person with an active disease to another person through the tiny droplets released into the air via coughs and sneezes. More importantly, not all people who harbor Mtb become sick, which would depend on the ability of their immune system to prevent the disease [118].

Although the number of newly diagnosed people with TB reported in 2020 was significantly below the average for 2019, due to the interruption of TB diagnostic services within the COVID restriction contexts, a global increase in the number of people who had died due to TB was registered [119]. This strongly highlights the fact that TB remains a persistent disease worldwide and a challenge in the medical research field.

Mtb can invade, grow, and proliferate within several host defense cells, including alveolar macrophages (AM), neutrophils, monocytes, and DCs [120]. For a wide range of pathogens, the phagocytic host cells recruited to the infection site contain or even eradicate the infection, but in the case of Mtb, the intervention of immune cells attracted to the infection site contributes to early bacterial growth, therefore leading to the formation of protective cellular aggregates called granulomas, which extends the cellular environment for Mtb expansion [121]. The ability to exploit the host’s immune system to its own advantage, the talent to manipulate the host cellular mechanisms for its replication [122], and the capability to arrest phagosome maturation [123] or phagosome–lysosome fusion [124] all make Mtb an extremely successful pathogen.

#### 2.3.1. Autophagy Activation in the Intracellular Elimination of Mtb

During Mtb infection, xenophagy is mainly triggered by the cytoplasmic release of Mtb. Indeed, through a type VII secretion system known as the ESAT-6 Secretion System-1 (ESX-1) virulence factor, Mtb mediates the phagosomal permeabilization that leads to its own release into the cytosol [125,126]. This event provides favorable circumstances for host cytosolic receptors to sense Mtb extracellular DNA. Cytosolic bacterial DNA can be sensed by both Stimulator of Interferon Response CGAMP Interactor 1 (STING) and the cytosolic DNA sensor, Cyclic GMP-AMP Synthase (cGAS), thus allowing for the targeting of Mtb through xenophagy [127]. In addition to xenophagy, NIX-mediated mitophagy has also been found to play an essential role in Mtb infection resolution [128]. In Mtb clearance, both the E3 ligase-dependent and -independent mechanisms have been reported. The secretion of the bacterial protein EsxA allows for the association of the host protein Ubiquilin 1 with Mtb, therefore promoting the IFN-γ-mediated autophagy clearance of Mtb [129]. In addition, the direct ubiquitination of cytosolic Mtb Rv1468c through a UBA-dependent interaction led to the recruitment of p62 to transfer Mtb into LC3-associated autophagosomes for Atg5-dependent autophagic clearance [41]. The E3 ubiquitin ligase Smurf has been found to be required for selective autophagy of Mtb and host defense against Mtb infection [130]. In addition, the presence of PARK2 has been reported to limit Mtb replication during acute Mtb infection [131]

#### 2.3.2. Exploiting Autophagy by Mtb

Notably, the successful pathogenesis inflicted by Mtb infection strongly relies on evading or exploiting the autophagy process; more importantly, the evolution of strategies by Mtb to avoid host autophagy highlights the critical importance of xenophagy in the fight against Mtb. It has been demonstrated in macrophages that the Mtb secretion of the Enhanced intracellular survival (Eis) protein plays crucial roles in the regulation of autophagy, inflammatory responses, and cell death [132]. In addition, Mtb is able to modulate the expression of several host miRNAs that play roles in the inhibition of autophagy, including miRNA-155, miRNA-133, miRNA-125a, miRNA-17-5p miRNA-144*, and miRNA-27a [133].

A recent work that employed the high-throughput loss-of-function screening of an Mtb transposon mutant library revealed that several Mtb proline-glutamate/proline-proline-glutamate (PE/PPE) proteins are involved in autophagy inhibition during Mtb infection [134]. Studies on DCs reported that Mtb infection may inhibit autophagy through the ESX-1 [135]. Particularly, Mtb inhibits RAB7 recruitment—a crucial step in autophagosome maturation; this occurs according to the virulence regulators PhoP and ESAT-6 [135]. Worthy of note is the fact that Mtb was proven to be insensitive to NADPH oxidase and the LAP process, which relies on the Mtb CpsA [136].

#### 2.3.3. Targeting Host Autophagy to Counteract Mtb Infection

Given the importance of autophagy in the control of Mtb infection, host-directed therapy (HDT) that targets autophagy represents an attractive adjunct to existing drugs directed at suppressing Mtb.

Interestingly, several compounds, such as Statins and Ambroxol, potentiate the effects of drugs used in Mtb treatment, inducing autophagy [137,138].

Over the years, in vitro and in vivo studies have revealed several promising candidates in host-adjunctive therapy for improving the effectiveness of TB treatment. The pharmacological induction of autophagy through the targeting of the AMPK/mTOR pathways has been reported to limit Mtb infection. Among them, the antidiabetic drug Metformin, acting in an AMPK-dependent manner, ameliorated the severity of infection in two human cohorts [139]. AMP-mimetic 5-aminoimidazole-4-carboxamide-1-β-D-ribofuranoside (AICAR), an AMPK-activating agent, and Ornithine have been found to induce autophagy through AMPK activation, leading to the inhibition of Mtb growth [139,140]. These studies indicate that intervention on AMPK could be a promising strategy to enhance autophagy and restrict Mtb survival.

Rapamycin was also reported to enhance autophagy and suppress the growth of intracellular Mtb [39]. Thus, Rapamycin analogs such as Everolimus could represent a potential HDT in the treatment of Mtb [141]. Baicalin, a herbal medicine, and Nilotinib, a tyrosine kinase inhibitor, have been reported to trigger autophagy in Mtb-infected macrophages through the inhibition of PI3K/Akt/mTOR [142,143]. In human macrophages, the anti-chronic lymphocytic leukemia drug, ibrutinib, has been found to inhibit Mtb growth and promote auto-lysosome fusion by inhibiting the BTK/Akt/mTOR pathway [144]. Bazedoxifene, a selective estrogen receptor modulator, also enhanced autophagy in Mtb-infected macrophages in a ROS-dependent manner, acting on Akt/mTOR signaling [145]. However, it is necessary to evaluate the specific context. Indeed, targeting the mTORC1 pathway with controlled Mtb infection to induce autophagy in HIV/Mtb co-infected patients could be detrimental for patients [146]. Interestingly, a very recent study found that trehalose promoted the reversion of an HIV-mediated autophagy block by promoting Transcription Factor EB (TFEB) nuclear localization. This event limited Mtb survival in co-infected HIV/Mtb cells and Mtb-infected animals, indicating that the activation of the TFEB nuclear translocation also represents a possible way to counteract Mtb infection [147].

Particularly noteworthy is the upregulation of autophagy by targeting the Purinergic Receptor P2X 7 (P2X7) through calcimycin; this has been reported to be involved in Mtb killing [148,149]. Accordingly, soybean lectin has been reported to induce autophagy in a P2X7R-NF-κB-ROS-dependent manner to kill intracellular Mtb [150].

All these results suggest that HDT targeting autophagy, in addition to drugs directed against Mtb, could represent an encouraging strategy for synergistic therapies to eliminate Mtb infection.

## 3. Autophagy in Lung Diseases

Increasing evidence highlights the importance of the role of autophagy and its selective forms in several lung diseases. In this section, we point out recent findings that implicate autophagy and the selective autophagic response in several human lung diseases, including Cystic Fibrosis (CF), Chronic Obstructive Pulmonary Disease COPD, and Malignant Mesothelioma (MM).

### 3.1. Autophagy in Cystic Fibrosis (CF)

CF is the most common hereditary disease in North America and Europe [151,152]. It is characterized by mutations in the Cystic Fibrosis Transmembrane Conductance Regulator (CFTR) gene, which cause multifunctional defects that lead to recurrent infections with consequent lung hyperinflammation and lung tissue damage in CF patients. The CFTR protein is a cAMP-activated and phosphorylation-regulated chloride channel, principally expressed to the apical membrane of epithelial cells. Among the defects associated with defective CFTR, perturbed calcium (Ca^2+^) signaling [153] plays a relevant role in the pathophysiology of CF lung disease [154,155]. In particular, the abnormal mitochondrial Ca^2+^ signaling exacerbates the airway inflammatory responses and affects the autophagy in CF [155,156]. Although the mechanisms linking the defective CFTR channel to altered autophagy remain unclear, autophagy is impaired in both the epithelial airways and immune cells of CF patients, with further repercussions on the expression, trafficking, and function of mutated CFTR channels [157,158].

#### 3.1.1. Dysregulation of Autophagy in CF

**Figure 3:**
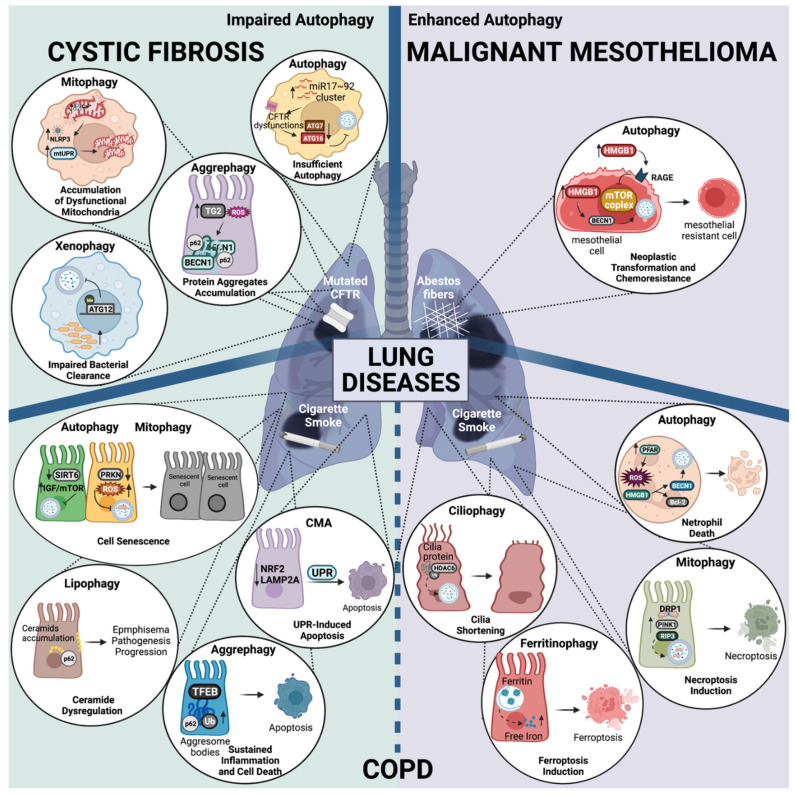
Autophagy dysregulation in lung disease progression. Representation of the main molecular mechanism underlying the role of autophagy pathway dysregulation, which is associated with Cystic Fibrosis (CF), Malignant Mesothelioma (MM), and Chronic Obstructive Pulmonary Disease COPD. On the left (green rectangle), the impairment of different autophagy pathways contributes to the exacerbation of pulmonary inflammation in CF (top) and COPD (bottom). Deficit in these degradation pathways lead to the accumulation of degradation products, which ultimately leads pathogenesis progression. On the right (violet rectangle), the enhancement of different autophagy pathways linked to COPD (bottom) and MM (top) are also illustrated inside the zoom circles. Although COPD progression has been associated with impaired autophagy, enhanced autophagy pathways have also been associated with the worsening of the disease. Increased autophagy in MM has been associated with neoplastic transformation and chemoresistance. The main cause of each disease is also reported: mutation in CFTR for CF, asbestos fibers for MM, and cigarette smoke for COPD. All the altered autophagy mechanisms underlying the above-mentioned lung diseases are extensively described in the main text. Created with BioRender.com.

Several autophagy proteins are downregulated in CF cells [159]. An in silico approach identified the elevated expression of the microRNA Mir17~92 cluster that targets autophagy proteins in CF cells, including macrophages [160] (Figure 3). By reducing the expression of Mir17 and Mir20, the CFTR functioning was ameliorated, thus mediating the restoration of autophagy and the re-expression of ATG7 and ATG16 [160].

The defective autophagy in CF airway cells is also associated with increased oxidative stress and the upregulation of Transglutaminase 2 (TG2), which mediates the sequestration of BECN1 and its accumulation in F508del-CFTR protein aggregates [157,159] (Figure 3).

In recent years, we have demonstrated that in CF airways during *P. aeruginosa* infection, increased ER-mitochondria juxtapositions favor an inter-organelle Ca^2+^ transfer via the Mitochondrial Calcium Uniporter (MCU) complex [161], which further downregulates the autophagy in CF, resulting in an augmented pathogen survival and the worsening of inflammatory responses [155,156]. CF airway cells exposed to pathogens showed the increased expression and interaction of the ER protein Vesicle-Associated Membrane Protein-Associated Protein B (VAPB) and the OMM Protein Tyrosine Phosphatase Interacting Protein 51 (PTPIP51), resulting in the tightening of tethers and the concomitant impairment of the selective autophagic responses, xenophagy and mitophagy [156]. The mitochondrial Ca^2+^-overload in CF cells led to persistent oxidative stress, mitochondrial unfolding protein response (UPR), and NLR Family Pyrin Domain Containing 3 (NLRP3) inflammasome activation, which contributed to the disturbance of selective autophagic responses, with detrimental repercussions on the capacity to sequester and eliminate invading pathogens or dysfunctional mitochondria (Figure 3). By controlling the mitochondrial Ca^2+^-overload via KB-R7943-dependent MCU inhibition, we rectified the selective autophagic responses that attenuate the *P. aeruginosa* hyperinflammation in CF lung both in vitro and in vivo, therefore restoring the bacterial clearance and preserving mitochondrial homeostasis [156].

It is commonly accepted that the recurrent infections in chronic CF lung pathology are a consequence of the excessive secretion of thick mucus, which becomes a culture medium for pathogens as it remains in the lungs and contributes to the persistent exacerbation of inflammation. New discoveries of defective autophagy in CF have led to the development of a new concept related to the susceptibility of CF patients to pathogen infections [162]. Recurrent infections are also the result of the defective xenophagic activity of CF immune cells, which, unable to sequester and destroy intracellular pathogens such as *P. aeruginosa* and *B. cenocepacia*, favor their chronic colonization in the lungs, surviving and persisting in the phagosomes of CF human cells [163,164,165,166]. In support of this, CF macrophages showed defective autophagy due to the reduced protein expression of ATG12 with respect to CFTR-wt immune cells; this was caused by the increased methylation of the *Atg12* promotor region, which favored *B. cenocepacia* replication [167] (Figure 3). The methylation of Atg12 was reduced by the natural compound epigallocatechin-3-gallate, which limited the dissemination of *B. cenocepacia* by rescuing autophagy in CF mice [167].

A higher amount of colony-forming (unit/mL) and interactions between xenophagic receptors as well as the invasion of *P. aeruginosa* were also observed in CF airway cells as compared to non-CF cells, indicating that the reduced bacterial clearance capacity is intrinsic to CF airways [156].

#### 3.1.2. Targeting Autophagy in CF

Preclinical studies have demonstrated the utility of autophagy restoration in controlling the pathogenesis and progression of CF lung disease. By regulating the mitochondrial Ca^2+^ overload via the MCU inhibitor KB-R7943, mitophagy and xenophagy were rectified both in vitro and in vivo, thus promoting bacterial clearance and inflammatory reduction in the CF airways [156].

The rescue of autophagy with cysteamine reduced the oxidative stress status and the release of pro-inflammatory cytokines in CF airway cells [157,168]. Cysteamine is an FDA-approved drug for nephropathic cystinosis that is able to ameliorate the maturation and trafficking of the F508del-CFTR channel to the plasma membrane, thereby enhancing the autophagic activity of CF airway cells [169]. The promising results of cysteamine or its derivates have been tested in phase-I/II trails, but the high doses required for the treatments and the evidence of side effects have limited their clinical usefulness [170,171,172]. Similarly, rapamycin has been shown to reduce lung inflammation, improving the CFTR function in a CF cell and mouse model [159,160]. The inhibitor of mTOR, which induces autophagy, achieved beneficial effects in CF. However, it again did not reach the patients due to several limitations in clinical practice, including huge pharmacokinetic variability, adverse side effects, and off-target effects [173,174,175,176].

Myriocin, an inhibitor of sphingolipid synthesis, induced autophagy in CF patient-derived monocytes mediating the up-regulation of genes involved in autophagy, lipid storage, and metabolism [177]. The profound transcriptional change promoted fungal clearance against *A. Fumigatus*. Another strategy to correct defective autophagy in CF is to enhance the levels of Nitric Oxide (NO). NO donors and/or S-Nitrosoglutathione (GSNO) inhibitors have been tested to rescue the autophagy in CF, with beneficial repercussions on the maturation and trafficking of CFTR and airway inflammation [178,179,180].

### 3.2. Autophagy in Chronic Obstructive Pulmonary Disease (COPD)

COPD is an inflammatory respiratory disease characterized by breathing problems and airflow limitations due to the impairment of lung functions [181,182]. Chronic inflammation is one of the main hallmarks in COPD that affects lung parenchyma and peripheral airways, resulting in airway remodeling characterized by detrimental changes in structural tissues along with the progressive and irreversible decline of lung functions [183]. Lung inflammation involves different cell types, the innate immune system cells, including epithelium cells [184] and inflammatory cells such as macrophages and neutrophils, as well as adaptive immune cells, mainly T lymphocytes [185]. Another important pathophysiological feature that contributes to COPD exacerbations is the presence of pro-inflammatory cytokines associated with mucociliary elevator dysfunction, mucus hypersecretion, and decreased mucus transport [186]. Although the major risk factor for COPD is tobacco smoke, significant exposure to environmental noxious particles or gases and genetic causes are also important determinants for this pathology [187,188,189]. Autophagy is an important homeostatic process involved in the regulation of inflammation [190]. A well-regulated autophagy process plays critical roles in the maintenance of lung function, which is why the dysregulation of different types of autophagy has been linked to the pathogenesis of COPD [191].

#### 3.2.1. Dysregulation of Autophagy in COPD

Autophagy in COPD may exert both protective and deleterious effects. Various studies have demonstrated that the activation of autophagy and its selective responses are linked to COPD progression. Indeed, an increase in the levels of several autophagic proteins has been observed in the lung tissue of COPD patients. Particularly, the exposure to cigarette smoke (CS) extract (CSE) induces an increase in LC3B expression in lung epithelial cells, which is mediated by an increase in the Early growth response-1 (Egr-1) and E2F transcription factors and by the reduction of HDAC activity. Accordingly, LC3-KO or Egr^−/−^ showed protective effects [192]. Soluble Epoxide Hydrolase (Ephx2) has also been reported to contribute to COPD pathology promoting autophagy activation [193]. Consistently, microRNA-21 has been found to worsen COPD pathogenesis by promoting autophagy in CSE-exposed human bronchial epithelial cells [194]. In addition, CS has been shown to induce autophagy in neutrophils by activating the Platelet-Activating Factor Receptor (PAFR); this event led to the increase of ROS and High Mobility Group Box 1 (HMGB1). Elevated HMGB1 interacted with BECN1, which led to its dissociation from Bcl-2 and promoted the assembly of autophagy and cell death, thus contributing to COPD progression [195] (Figure 3). Particularly noteworthy is that the bioinformatic analysis identified 40 potential autophagy-related genes in COPD patients. When the blood samples from COPD patients and healthy controls by qRT-PCR were compared, an increase in the RNA expression of the autophagy-related genes *HIF1A, CDKN1A, BAG3, ERBB2,* and *ATG16L1* was found [196].

Similar to autophagy, enhanced mitophagy has also been associated with the pathogenesis of COPD, probably depending on the extent of the damage. In the lung epithelial cells of COPD patients, increased PINK1 and Receptor Interacting Serine/Threonine Kinase 3 (RIP3) expression have been reported. In vitro and in vivo results suggest that mitophagy-dependent necroptosis in COPD lungs is enhanced in response to CS exposure; accordingly, Pink1^−/−^ and Mdivi-1-treated mice showed decreased mitochondrial dysfunction and mucociliary clearance disruption during CS exposure [197] (Figure 3). Increased mitochondrial fission, mitophagy, and mitochondrial oxidative stress, have been reported in lung cells upon CS exposure [198,199]. In this context, it was found that CSE-treated HBECs and COPD mice expressed high levels of FUNDC1. In addition, FUNDC1 interacted with Dynamin 1 Like (DRP1), inducing mitophagy in CSE-treated cells (Figure 3). Consistently, FUNDC1 or DRP1 silencing inhibited mitophagy and apoptosis, thus attenuating COPD [200]. Similarly, NIX-dependent mitophagy has been reported to be involved in COPD pathogenesis by promoting airway epithelial cell and mitochondria injury [201].

Intriguingly, COPD has been associated with cilia shortening and mucociliary clearance (MCC) disruption; autophagy, and more specifically, ciliophagy (the consumption of cilia components by autophagy) has been reported to be involved in these processes [202,203]. Particularly, Histone Deacetylase 6 (HDAC6) hypomethylation and increased protein expression have been found in human COPD. More importantly, it has been proposed that cytosolic HDAC6 induces the autophagy-mediated cilia shortening and MCC disruption during CS exposure [204] (Figure 3). In addition, Nuclear Receptor Coactivator 4 (NCOA4)-mediated ferritin selective autophagy (ferritinophagy) in response to CS treatment has been observed in COPD as a consequence of the altered homeostasis in iron; in fact the CS-induced ferroptosis plays a critical role in COPD pathogenesis [204] (Figure 3).

However, while these results undoubtedly demonstrate that autophagy and several selective forms of autophagy support COPD pathology, there are also a lot of evidence of impaired processes in COPD. COPD-emphysema exacerbations have been linked to autophagy impairment caused by CS [205]. In HBECs from COPD patients, CSE exposure led to the insufficient autophagic clearance of damaged proteins inducing cell senescence. These results suggest a protective role for autophagy in COPD pathology [206]. In this regard, Sirtuin 6 (SIRT6) attenuated the IGF-Akt-mTOR signaling, therefore promoting CSE-induced HBEC senescence via autophagy regulation [207] (Figure 3). Autophagy activation has been shown to be important in COPD patients infected by the human rhinovirus that potentiates antiviral and the anti-inflammatory activities [208].

Lungs require a high amount of energy in order to adapt their respiratory function, so it is not surprising that mitochondria quality control is important for lung homeostasis. As for autophagy, insufficient mitophagy has been associated with COPD pathogenesis. Indeed, several works reported that impaired PINK1-PARK2-mediated mitophagy promoted CS stress-induced lung cellular senescence (Figure 3), suggesting that the restoration of mitophagy delayed cellular senescence [209,210,211].

Impaired lipophagy in COPD-emphysema has been suggested to be implicated in ceramide accumulation. Indeed, the CS exposure-induced sphingolipid imbalance promoted the accumulation of membrane and intracellular-ceramide into p62 positive aggresome bodies sustaining the COPD-emphysema pathogenesis [212] (Figure 3).

Interestingly, BEAS-2B cells and C57BL/6 mice exposed to either cigarette smoke extract (CSE) or a sub-chronic CS treatment showed defective autophagy in COPD-emphysema through the induction of the perinuclear localization of TFEB to aggresome bodies [213]. Coherently, elevated p62 accumulation in smokers with severe COPD-emphysema lungs confirmed the pathogenic role of autophagy impairment in aggresome formation and in COPD-emphysema progression (Figure 3). Consistently, autophagy induction by carbamazepine inhibited CS-triggered aggresome formation in BEAS-2B cells [213]. Thus, this indicates that in the proteostasis imbalance, which characterizes the COPD pathology, the selective disposal of aggregates by aggrephagy is affected.

Besides autophagy and selective autophagy, the importance of CMA in COPD pathology has also been reported. More importantly, CS has been found to induce the accumulation of misfolded proteins and enhance the Unfolded Protein Response (UPR), leading to apoptosis in COPD pathogenesis [214,215]. In this context, a functional crosstalk between UPR and CMA has been reported [216]. CSE treatment attenuated LAMP2A in HBECs, leading to increased apoptosis. CMA activity may be involved in the mechanism for epithelial cell apoptosis by enhancing CS-induced UPR in COPD pathogenesis [217]. Consistently, a recent work reported reduced levels of NFE2 Like BZIP Transcription Factor 2 (Nrf-2) and LAMP2A in the small airway epithelial cells of COPD patients with respect to non-COPD lungs, indicating that impaired CMA is associated with COPD pathogenesis through the enhancement of UPR-mediated apoptosis [218] (Figure 3).

The discrepancies between the results of various studies on the role of different forms of autophagy in COPD do not have a unifying explanation, which reflects the complex roles of these degradative pathways in the COPD pathology.

#### 3.2.2. Targeting Autophagy in COPD

With regard to COPD contexts, autophagy modulation through induction or inhibition has been reported to have beneficial effects in COPD pathology. In COPD cases associated with impaired autophagy, the induction of this process counteracted COPD pathology. Indeed, Curcumin promoted autophagy, inhibited ER stress, and reduced the pro-inflammatory cytokine levels, thus alleviating COPD through SIRT1-dependent activation [219]. Leukotriene B4 (LTB4), a pro-inflammatory mediator, is involved in the chronic inflammation in COPD. Treatment with the BLT1 antagonist to inhibit the LTB4/BLT1 signaling pathway ameliorated the inflammatory response through the autophagy induction of CS-exposed macrophages [220]. Myotubularin-related protein 14 expression was reduced in COPD patients, its overexpression improved mitophagy and inhibited CSE-induced inflammation and apoptosis in both in vitro and in vivo models [221]. Autophagy induction by carbamazepine, an approved autophagy-inducing drug, reduced aggresome formation and alveolar space enlargement in BEAS-2B cells treated with CSE [222]. In BEAS-2B cells treated with waterpipe smoke extract or nicotine showed ubiquitinated cytosolic protein accumulation. Treatment with Gemfibrozil, a potent TFEB-inducing autophagic drug, protected from CS-induced TFEB/autophagy-impairment and COPD-emphysema pathogenesis [213]. Treatment with cysteamine, an autophagy inducer and an antioxidant drug, rescued aggresome formation by inducing aggrephagy [223]. In addition, cysteamine treatment has been reported to induce lipophagy, which attenuates ceramide accumulation and thus COPD-emphysema pathogenesis [212].

On the other hand, the detrimental consequences of CS exposure-activated autophagy could be prevented by inhibiting autophagy processes. About this, 14,15-Epoxyeicosatrienoic acid, a metabolic product of free arachidonic acid with anti-inflammatory effects, has been reported suppress CS condensate-induced inflammation in lung epithelial cells by promoting the accumulation of Nrf-2 via the inhibition of autophagy [224]. Particulate matter-induced experimental COPD has been shown to promote the aberrant expression of NADH dehydrogenase genes and enhanced autophagy. Treatment with taurine and 3-methyladenine ameliorated the emphysema inhibiting the autophagy by restoring mitochondrial gene expression. Consistently, ATG7-deficient mice were protected from particulate matter (PM)-induced experimental COPD [225]. CS-induced autophagy in synergy with the ERK/MAPK pathway has been found to produce excessive inflammation, while treatment with Silymarin attenuated the inflammatory responses intervening in the crosstalk between autophagy and the ERK/MAPK pathway [219].

The treatment of epithelial cells with Quercetogetin has been proved to inhibit CSE-induced mitophagy and cell death by reducing the phospho-DRP-1 levels, thus suppressing apoptosis [226]. In the human epithelial cell Puerarin, the inhibition of FUNDC1-mediated mitophagy through the activation of the PI3K/AKT/mTOR signaling pathway reduced CSE-induced apoptosis [227]. Lastly, Roflumilast, a phosphodiesterase 4 Inhibitor, has been found to protect epithelial cells from CS-induced mitophagy-dependent cell death [228].

### 3.3. Autophagy in Malignant Mesothelioma (MM)

MM is a fatal and aggressive neoplasm of the serosal cavities for which there are no effective therapies. Patients with MM have a 50% chance of survival 15 months after diagnosis, and it has been estimated that approximately more than 40,000 deaths per year worldwide are caused by MM. Different factors are involved in the onset and development of MM [229]. Throughout the years, evidence has shown that MM is primarily associated with exposure to carcinogenic mineral fibers, particularly asbestos. Once inhaled, asbestos settles in the pleura where it provokes severe damage to the mesothelial cells. At the beginning of exposure, asbestos induces the apoptotic and necrotic death of mesothelial cells, which then release pro-inflammatory mediators in the extracellular space. Among them, the most important is the proinflammatory protein HMGB1, whose activation induces the inflammatory response. Regrettably, cells do not have the ability to remove the asbestos fibers [230]. Therefore, the asbestos-induced inflammatory process self-propagates and sustains the creation of a chronic inflammatory environment characterized by presence of mutagenic factors, which lead to the malignant transformation.

#### 3.3.1. Dysregulation of Autophagy in MM

Recently, it has been proven that autophagy plays a pivotal role in this HMGB1-mediated transformation.

Indeed, human primary mesothelial cells exposed to different carcinogenic fibers displayed increased autophagy accompanied by the release and activation of HMGB1. Particularly, it has been proposed that extracellular HMBG1 acts on the RAGE-mTOR-ULK pathway while cytosolic HMGB1 acts on BECN1, thus promoting autophagy induction (Figure 3). The use of the genetic interference of HMGB1 or a conditional HMGB1-KO mouse model showed that the loss of HMGB1 reduced asbestos-induced autophagy and the consequent cellular transformation [231] (Figure 3). Furthermore, by using the antidepressant drug desmethylclomipramine as an autophagy inhibitor, it was possible to interfere with the asbestos-mediated cell transformation [231]. Autophagy was found to be a critical event for the MM onset; however, this catabolic mechanism was also found to be critical for its progression since increased levels of autophagy were found in MM [232,233]. In addition, autophagy elicits resistance towards several chemotherapy interventions in MM [232].

#### 3.3.2. Targeting Autophagy in MM

Overall, these findings suggest that autophagy may be a reliable target in MM therapy. Consistently, a concomitant blockage of autophagy and Bcl-2 homology domain-3 (BH3) mimetics (commonly used for the treatment of several cancers) reduced the MM growth and increased the apoptosis [234]. In line with this, the specific inhibitor of ULK1, MRT 68921, reduced the excessive autophagy of MM cells and potentiated the efficacy of chemotherapy to kill cells [235]. In addition, the combination of autophagy inhibitor bafilomycin 1 with the isothiocyanate compound (derived from glucoraphanin sulforaphane and cisplatin) enhanced the cell death of MM cells [236]. Pemetrexed, a multitargeted antifolate cytotoxic agent, represents the first line of intervention against MM. Different enzymes have been described as pemetrexed targets. Among them, it has been demonstrated that this chemotherapy agent may activate apoptosis in MM cells by downregulating class III NAD^+^-dependent deacetylases-sirtuins (SIRTs) activities. Consistent with this, the use of resveratrol, an activator of SIRTs, reduced the efficacy of pemetrexed to kill tumor cells. A recent work suggested that SIRTs activities were also crucial for the regulation of the autophagy process in MM during stress conditions induced by chemotherapy (such as DNA damage and oxidative stress), thereby providing other evidence of the importance of autophagy in controlling the response to chemotherapy in MM [237].

In recent years, several investigations have unveiled other important factors for MM onset. Among them, the germline mutations of the tumor suppressor BRCA1 Associated Protein 1 (BAP1) and the exposure to Simian Virus 40 (SV40) are considered to be the main contributors [238,239], although there is no direct evidence that the presence of BAP1 mutations or SV40 infection modulates autophagy to regulate the development of MM.

## 4. Conclusions

Autophagy plays multifaced roles in lung infections and lung disease progression. It can have both harmful and protective roles. The complexity of the function of autophagy in these conditions highlights the difficulties in adopting a unifying mechanism that would modulate autophagy for an effective therapeutic intervention, and above all, it highlights the urgency of further research to develop optimized strategies that are capable of manipulating autophagy in a context-dependent manner. More importantly, further research to define the contribution of other autophagic pathways in lung disease, including selective forms of autophagy, chaperone-mediated autophagy, and microautophagy, would shed light on potential targets for novel and valid therapeutic interventions.

## Data Availability

Not applicable.

## Abbreviations

AICAR	AMP-mimetic 5-aminoimidazole-4-carboxamide-1-β-D-ribofuranoside
AM	Alveolar Macrophages
AMBRA1	Activating Molecule in BECN1-Regulated Autophagy protein 1
AMPK	5′ AMP-Activated Protein Kinase
ATGs	Autophagic-Related Genes
BAP1	BRCA1 Associated Protein 1
BECN1	Beclin 1
BNIP3	Bcl-2 Interacting Protein 3
BNIP3L/NIX	Bcl-2 Interacting Protein 3 Like
CF	Cystic Fibrosis
CFTR	Cystic Fibrosis Transmembrane Conductance Regulator
cGAS	cyclic GMP-AMP synthase
CMA	Chaperone-Mediated Autophagy
COPD	Chronic Obstructive Pulmonary Disease
CS	Cigarette smoke
CSE	Cigarette smoke extract
DCs	Dendritic cells
DFCP1	Zinc Finger FYVE-Type Containing 1
DRP1	Dynamin 1 Like
Egr-1	Early growth response-1
Eis	Enhanced intracellular survival
Ephx2	Soluble epoxide hydrolase
ER	Endoplasmic Reticulum
ESCRT	Endosomal Sorting Complexes Required for Transport
ESX-1	ESAT-6 Secretion System-1
ESX-1	ESAT-6 Secretion System-1
FAM134B	Family With Sequence Similarity 134 Member B
FIP200	RIB-inducible coiled-coil protein 1
FUNDC1	FUN14 Domain Containing 1
GABARAP	Gamma-Aminobutyric Acid Receptor-Associated Protein
GSNO	S-Nitrosoglutathione
HDAC6	Histone Deacetylase 6
HDT	Host directed therapy
HMGB1	High Mobility Group Box 1
HSC70	Heat Shock Cognate 71 kDa Protein
IFN	Interferon
IL	Interleukin
LAMP2A	Lysosome-Associated Membrane Protein type 2A
LAP	LC3-associated phagocytosis
LC3	Microtubule-Associated Protein 1A/B Light-Chain 3
LIR	LC3-Interacting Region
LTB4	Leukotriene B4
MAVS	Mitochondrial Antiviral-Signaling Protein
MCC	Mucociliary clearance
MCU	Mitochondrial Calcium Uniporter
Mtb	*Mycobacterium tuberculosis*
mTOR	Mechanistic target of rapamycin
NBR1	Neighbor of BRCA1 Gene 1 Protein
NCOA4	Nuclear Receptor Coactivator 4
NDP52	Calcium Binding And Coiled-Coil Domain 2
NLRP3	NLR Family Pyrin Domain Containing 3
NO	Nitric Oxide
NOX2	NADPH oxidase-2
Nrf2	NFE2 Like BZIP Transcription Factor 2
OMM	Outer Mitochondrial Membrane
OPTN	Optineurin
ORF	Open Reading Frame
P2X7	Purinergic Receptor P2X 7
p62/SQSTM1	Sequestome
PAFR	Platelet-Activating Factor Receptor
PAMPs_viral	Pathogen-Associated Molecular Patterns
PARK2	Parkin RBR E3 Ubiquitin Protein Ligase
PE	Phosphatidylethanolamine
PI3KC3	Phosphatidylinositol 3-Kinase Catalytic Subunit Type 3
PI3P	Phosphatidylinositol 3-Phosphate
PINK1	PTEN Induced Kinase 1
PM	Particulate Matter
PTPIP51	Protein Tyrosine Phosphatase Interacting Protein 51
QF	Qingfei
RAB7	Ras-Associated Protein 7
RIP3	Receptor Interacting Serine/Threonine Kinase 3
ROS	Reactive Species of Oxygen
RSV	Respiratory Syncytial Virus
Rubicon	RUN And Cysteine Rich Domain Containing Beclin 1 Interacting Protein
SARS-CoV-2	Severe acute respiratory syndrome coronavirus–2
SIRT	Sirtuin
SKP2	S-Phase Kinase Associated Protein 2
SNAP-29	Synaptosome Associated Protein 29
SNARE	Soluble N-ethylmaleimide Sensitive Fusion Protein (NSF) Attachment Receptor
STING	Stimulator of Interferon Response CGAMP Interactor 1
STX17	Syntaxin 17
TAX1BP1	Tax1 Binding Protein 1
TB	Tuberculosis
TFEB	Transcription Factor EB
TG2	Transglutaminase 2
ULK1	Unc-51-Like Kinase 1
UPR	Unfolding protein response
UVRAG	UV radiation resistance-associated gene protein
VAMP-8	Vesicle Associated Membrane Protein 8
VAPB	Vesicle-Associated Membrane Protein-Associated Protein B
VPS	Vacuolar Protein Sorting
WIPI2	WD Repeat Domain, Phosphoinositide Interacting 2
